# Parent-Adolescent Communication on Sexual and Reproductive Health Issues and Associated Factors among Preparatory and Secondary School Students of Dabat Town, Northwest Ethiopia

**DOI:** 10.1155/2020/4708091

**Published:** 2020-07-25

**Authors:** Nigussie Dagnachew Adam, Getu Debalkie Demissie, Abebaw Addis Gelagay

**Affiliations:** ^1^World Vision International, Addis Ababa, Ethiopia; ^2^Department of Health Education and Behavioral Sciences, Institute of Public Health, College of Medicine and Health Sciences, University of Gondar, Gondar, Ethiopia; ^3^Department of Reproductive Health, Institute of Public Health, College of Medicine and Health Sciences, University of Gondar, Gondar, Ethiopia

## Abstract

**Background:**

A significant number of adolescents as well as young men and women aged 10 to 24 years die each year in the world due to avoidable sexual and reproductive health problems such as unwanted pregnancy, unsafe abortion, and sexually transmitted infections, including HIV/AIDS. This is attributed to low access to and/or uptake of sexual and reproductive health services.

**Objective:**

To assess parent-adolescent communication on sexual and reproductive health issues and associated factors among secondary and preparatory school students in Dabat town, northwest Ethiopia, 2018.

**Methods:**

An institution-based cross-sectional study was conducted from April 1 to 10, 2018, using multistage sampling technique. Data were collected from 550 participants using structured, pretested, and self-administered questionnaire; entered into Epi Info version 7; and analyzed using SPSS version 20. Both bivariate and multivariable logistic regression analyses were employed, and variables with less than 0.05 *P* value in the multivariable regression were considered as statistically significant. Adjusted odds ratio with 95% CI was used to determine the strengths and directions of associations.

**Result:**

This study revealed that 48.5% of the participants discussed sexual and reproductive health issues with parents. Male sex (AOR = 1.6; 95% CI: 1.1–2.25), family income greater than ETB 1,000 (AOR = 1.6; 95% CI: 1.02–2.57), good knowledge of sexual and reproductive health (AOR = 1.5; 95% CI: 1.03–2.09), and favorable attitude to sexual and reproductive health issues (AOR = 1.9; 95% CI: 1.29–2.67) were factors significantly associated with parent-adolescent communication on the issues.

**Conclusion:**

This study showed that the proportion of parent-adolescent communication on sexual and reproductive health issues was low. Male sex, family income greater than ETB 1,000, and good knowledge and favorable attitude of adolescents had significant association with the communication.

## 1. Introduction

Adolescence is the period between 10 and 19 years marking a continuum of physical, cognitive, behavioral, and psychosocial changes characterized by increasing levels of individual autonomy and a growing sense of identity and self-esteem [1]. Our world is home to 1.2 billion adolescents, 70% of whom live in developing countries. Currently adolescents in Ethiopia constitute over 24 million (20%) of the total population. The adolescents of mainly sub-Saharan Africa are disproportionately affected by HIV/AIDS [2–4]. The survival, health, and well-being of adolescents are essential for ending extreme poverty, promoting development and resilience, and achieving all of the Sustainable Development Goals (SDGs). They are the most powerful agents for improving their own health and building prosperous and sustainable societies [5].

Globally, a significant number of adolescents and youth aged 10 to 24 years die each year due to lack of information about available health services and avoidable sexual and reproductive health negative consequences, such as unwanted pregnancy, unsafe abortion, and sexually transmitted infections including HIV/AIDS. This is attributed to poor access to, and low uptake of sexual and reproductive health services. At present, the health of adolescents is increasingly at risk owing to urbanization and change in life style. HIV/AIDS and other reproductive health problems are no doubt the greatest threats to their well-being [6, 7].

Parent-adolescent communication is defined as a fundamental process through which parents convey ideas, values, beliefs, expectations, information, and knowledge to their children [1]. The frequency of parent-adolescent communication about sexual issues is the most common concept used to study parental impact on adolescent sexual attitude and behavior. For example, the more parents discuss topics such as sexual relationship, pregnancy, and sexually transmitted infections (STIs) including acquired immune deficiency syndrome (AIDS) with their adolescents, the less likely the adolescents will be engaged in risky sexual behavior, and the more likely they will delay their first sexual act [8]. A systematic review of behavioral studies found that family connectedness and general and sexuality-specific parent-young people discussions had a protective association with youth sexual and reproductive health outcomes [9].

Parent-child communication is limited by cultural barriers and parents' lack of knowledge about sexual and reproductive health issues [10]. Many adolescents discuss SRH issues with their peers (who may or may not have proper knowledge of these matters); as a result, they gain patchy knowledge. This misinformation can make adolescents vulnerable to unprotected sex, unwanted pregnancies, sexually transmitted diseases, and unsafe abortions [11].

The main barrier to communication between parents and adolescents is the fact that they belong to two different generations. Parents are often unable to keep pace with changing social and technical developments, so that they fail to understand their adolescent children. At the same time, adolescents cannot understand the mindset of their parents who, as they feel, have a very traditional outlook [12].

Previous studies on premarital sex in Ethiopia showed that a significant proportion of school adolescents (24.8% in the eastern and 21.4% in the northern part of the country) were sexually active [13]. Male and female adolescents in the region access sexual education from schools through health clubs, mini media, and community health workers. Still 60% of adolescent pregnancies are unwanted, resulting from unprotected sexual intercourse among the youth, 1.1% of whom are infected with HIV [14].

A study conducted in Ethiopia showed that although most of the parents said that discussion about sexual and reproductive health between adolescents and parents was important, it was indicated that there were gaps in discussing the positive aspects of adolescent sexuality [15]. Parent-adolescent communication about sexuality has been controversial issue. Most parents do not feel comfortable to talk with their adolescents about sexual issues and tend to limit conversations to safe topics [16].

In fact, factors affecting parent-adolescent discussions should be taken into account during planning of SRH programs though there is little information about level of the communication and associated factors in the region. Therefore, this study was conducted to assess factors that affect parent-adolescent discussions on SRH issues among secondary (grades 9-10) and preparatory (grades 11-12) school students in Dabat town, northwest Ethiopia.

## 2. Methods

### 2.1. Study Setting and Period

The study was conducted at Dabat town high and preparatory school from April 1 to 10, 2018. The town is found in North Gondar zone, the Amhara Regional State located 800 km away from Addis Ababa, the capital of Ethiopia. It has an estimated population of 182,273, 50.5% of whom were male. Dabat had three elementary, one secondary, and one preparatory schools with 4,120 students in the 2018/2019 academic year. Of these, 54.5% were female.

### 2.2. Study Design, Participants, and Sample Size Determination

A school-based cross-sectional study was conducted on grade 9 to 12 secondary and preparatory school students in 2018. The school had a total of 4,120 students in the 28, 27, 9, and 6 sections of grade 9, 10, 11, and 12, respectively. All healthy students aged 15–19 years were included in the study, while those who were sick were excluded.

Sample size was calculated using the single population proportion formula considering the following assumptions: 68.2% of students communicating with parents [17], 5% margin of error, 95% confidence level, 1.5 design effect, and 10% nonresponse rate that yielded a sample size of 550.

### 2.3. Sampling Technique and Data Collection Procedures

The participants were selected using the multistage sampling technique. Initially, students were stratified from grades 9 to 12; then, from each grade, sections and participants were selected using the simple random sampling method. To select the participants, student rosters were used as sampling frames, and to determine the number of students in each grade, proportional allocation was employed.

Data were collected using pretested, structured, and self-administered questionnaire based on literature which comprised sociodemography, knowledge, and attitude of adolescent-parent communication components. The tool was first prepared in English and translated into Amharic and then back to English for the purpose of consistency. Data were gathered by six collectors and two supervisors who were trained for a day. The purpose and objectives of the study were explained to the participants ahead of the process.

### 2.4. Measurement

- Parent-adolescent communication on SRH issues: students who discussed at least two of the SRH issues (contraception, STIs/HIV/AIDS, sexual intercourse, unwanted pregnancy, avoiding premarital sex, condom, changes during puberty, and menstrual cycle) were considered to have communicated on SRH issues.
- Good knowledge of SRH: students who scored above the mean, 2.39 (SD ± 1.11), from the four general questions which assess knowledge were considered to be knowledgeable.
- Favorable attitude towards SRH: students who scored above the mean, 3 (SD ± 1.08), in the five items which assess attitude were considered to have favorable attitude.

### 2.5. Data Management and Analysis

The collected data were entered into Epi Info version 7.0 and exported to SPSS version 20 for analysis. Data were checked for any missing values and cleaned for completeness and errors. All variables with a *P* value of ≤0.2 in the bivariate analysis were further analyzed using the multivariate logistic regression model. Variables with *P* values <0.05 in the multivariate analysis were considered as significant predictors.

## 3. Results

### 3.1. Sociodemographic Characteristics of Respondents

A total of 550 school adolescents were enrolled in the study with 100% response rate. About 280 (50.9%) of the participants were rural dwellers and 320 (58.2%) were females. The mean age of the students was 17.6 (SD ± 0.96) years; 432 (78.5%) of them lived with their mothers and fathers (Table 1).

### 3.2. Source of Information on SRH Issues for Adolescents

**Figure 1:**
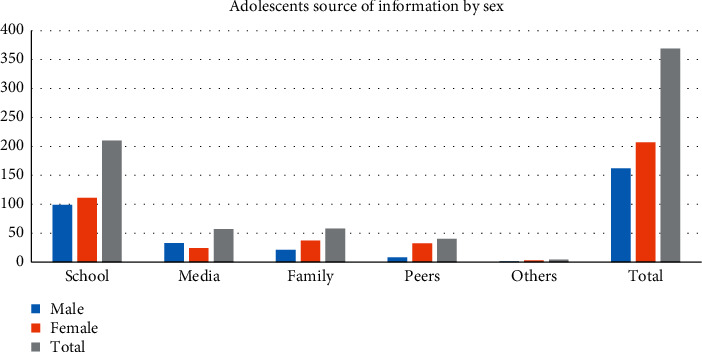
Source of information on SRH issue among secondary and preparatory students, Dabat town, Northwest Ethiopia, 2018.

Of the participants, 369 (67%) heard about SRH issues from different sources. School was the major source of information for 75% of respondents (Figure 1).

### 3.3. Sexual Attitude and Behavior of Adolescents

Of the respondents, 416 (75.6%) believed that parent-adolescent communication delays first sex; 102 (18.5%) held that it was normal and acceptable to have sexual feeling during adolescence; 448 (81.4%) disapproved premarital sex; 167 (30.4%) had sexual intercourse, and the mean age for adolescents to have first sex was found to be 17 years (SD ± 1.28). The most predominant reason for sex was love for 110 (65.8%) and peer pressure for 47 (28.5%); 83 (49.7%) made love to school class mates and 21% to casual acquaintances.

Eighty-five (51%) of the sexually active adolescents used condoms during their first sexual exposure and 8 (1.5%) developed sexually transmitted diseases because of not utilizing condoms; 32 (10%) of the female respondents reported they had pregnancy, 14 (44%) of which were unwanted; 10 (31%) and 2 (15.4%) faced abortions and preterm births, respectively. The majority, 69 (12.5%), of the participants took addictive substances, mainly alcohol 60 (87%), while 5 (7.2%) and 4 (5.8%) used chewing khat and cigarettes, respectively.

### 3.4. Parent-Adolescent Communication on SRH

Although 69% of the respondents reported that it was important to discuss sexual and reproductive health issues with parents, only 48.5% had communicated with their parents. Parent preferences for discussions depend on the types of topic. For instance, on premarital sex, 57.8% of the males preferred to talk to their fathers, while 71.8% of the females opted to talk to their mothers (Table 2).

### 3.5. Factors Associated with Parent-Adolescent Communication

Bivariate and multivariable analysis were done to assess whether there was any association between parent-adolescent communication and other independent variables. Sociodemographic variables, knowledge of SRH issues, attitude to such issues, and sexual behavior related variables were considered in the bivariate analysis. The result of the multivariable analysis showed that male sex, family income of greater than ETB 1,000, good knowledge of SRH issues, and favorable attitude towards SRH issues were significantly associated with parent-adolescent communication on the issues.

The odds of parent-adolescent communication were 1.6 times [AOR = 1.6; 95% CI: 1.1–2.25] higher among male students than females. The odds of parent-adolescent communication were 1.6 times [AOR = 1.6; 95% CI: 1.0–2.57] higher among secondary and preparatory school students whose families had monthly income of >ETB 1,000.

The odds of parent-adolescent communication were 1.5 times [AOR = 1.5; 95% CI: 1.03–2.09] higher among students who had good knowledge of SRH issues than those who had poor knowledge. The odds of having parent-adolescent communication were 1.9 times [AOR = 1.9; 95% CI: 1.29–2.67] higher among students who had favorable attitude towards SRH issues than their counterparts (Table 3).

## 4. Discussion

The aim of this study was to assess the level of parent-adolescent communication on sexual and reproductive health issues and associated factors in Dabat town, northwest Ethiopia, 2018. The study revealed that only 48.5% of the respondents aged 15–19 years discussed sexual and reproductive health issues with parents showing that SRH related topics continued to be sociocultural taboos between young people and their parents.

This finding was lower than that of a systematic review conducted in South Africa and found that approximately three-quarters of the students talked about HIV/AIDS with parents [18]. However, this finding was higher than those of other studies done in Ethiopia (Debre Markos (36.9%), Boditi (40.7%), and Dire Dawa (37%) [6, 19, 20]). The difference might be due to demographical and cultural variations in accessing SRH information, indicating that parent-child communication was affected by cultural barriers and parents' lack of knowledge about sexual and reproductive health matters.

The greatest proportion of parents participated in discussion about STIs, mainly HIV/AIDS. This finding was similar to those of other studies conducted in Ethiopia [21, 22]. Even parents who never talked about other SRH issues with their children mentioned that they discussed HIV/AIDS. This was because HIV/AIDS was considered a catastrophic disease that interfered with the family economic resources and family lineage by killing adolescents before they reach adulthood.

In this study, students preferred to talk with mothers, and similar results were reported by studies done in Ethiopia (Benishangul-Gumuz, Wello, and Harar) [15, 17, 23] and other countries [24–26]. The reason may be that the majority of mothers were housewives who had enough time to talk with their children. Moreover, the difference could be due to the closeness of adolescents to mothers than fathers as reported by another study [25].

School and the mass media were preferred sources of sexual and reproductive health information. That was similar to what was noted in north eastern Ethiopia [17].This may suggest that there is a need to equip school mates (peers) and the mass media with appropriate information and materials on SRH issues.

This study found that male students were more likely to communicate on SRH issues than females as recorded by other studies [15, 27]. This might be due to the fact that males are not heavily influenced by social taboos and tend to overcome cultural beliefs than females. Moreover, parental monitoring is strong on females than males, and such strict monitoring may limit open discussions on SRH issues with parents.

In this study, family income was found to be one of the significant factors associated with parent-adolescent communication on SRH-related issues. This is similar to findings by other studies [17, 21, 26]. This might be due to the fact that parents/caretakers with low income find it difficult to access radio, TV, and print materials for SRH information. They might be also likely to be less close to their children, which curtails the communication process as documented by another study [28]. Other finding also showed that there was lack of communication on sexuality issues between families in poor socioeconomic status and adolescents [29].

This study also revealed that adolescents who had knowledge of and favorable attitude to sexual and reproductive health related matters were more likely to discuss the issues with their parents than their counterparts. This is in line with other studies [17, 19, 21]. Lack of knowledge and poor attitude are communication barriers that make parents less confident or skeptical of talking about sexual topics and responding to the concerns raised by adolescents and ashamed to talk about sex related issues [30, 31]. Moreover, the study implied that adolescents were poorly informed by their parents about sexual matters, resulting in their being at high risk of contracting STDs including HIV/AIDS, unwanted pregnancies, and consequences such as abortion and school dropouts.

## 5. Conclusion

This study showed that the majority of the respondents had no discussion with their parents. Male sex, family monthly income of greater than ETB 1,000, and good knowledge of and favorable attitude towards SRH matters were significantly and positively associated with parent-adolescent communication. Though there are different potential sources of SRH information for adolescents in Ethiopia including families/parents, school clubs, social media, and peers, more than half of the respondents had no adequate knowledge regarding SRH issues. In a situation where such contributions are low, it is important that the health sector uses community health workers (health extension workers in Ethiopian context) to approach parents and encourage them to discuss the issues with their adolescents.

## Figures and Tables

**Table 1:** Sociodemographic characteristics of secondary and preparatory school students of Dabat town, northwest Ethiopia, 2018 (*n* = 550).

Variable		Male no. (%)	Female no. (%)	Total no. (%)
Age	15–17	85 (38.2)	137 (61.8)	222 (40.3)
	18-19	145 (44.2)	183 (55.8)	328 (59.7)

Grades	9-10	189 (41.3)	269 (58.7)	458 (83.3)
	11-12	41 (44.6)	51 (55.4)	92 (16.7)

Residence	Rural	120 (42.9)	160 (57.1)	280 (50.9)
	Urban	110 (40.7)	160 (59.3)	270 (49.1)

Religious participation	Every day	147 (41.4)	208 (58.6)	355 (64.5)
	At least once a week	59 (45.0)	72 (55.0)	131 (23.8)
	At least once a month	16 (35.6)	29 (64.4)	45 (8.2)
	Once a year	6 (35.3)	11 (64.7)	17 (3.0)
	Never attending	2 (100)	0 (0)	2 (0.5)

Living condition	With both parents	178 (41.2)	254 (58.8)	432 (78.5)
	With mother/father only	23 (37.7)	38 (62.3)	61 (11.0)
	With relatives	10 (43.5)	13 (56.5)	23 (4.2)
	Living alone	19 (55.9)	15 (44.1)	34 (6.3)

**Table 2:** Adolescent students' topics of discussions with their parents and families about SRH in Dabat town, northwest Ethiopia, 2018 (*n* = 550).

Topic of discussion	Father		Mother		Both father and mother	
	Male no. (%)	Female no. (%)	Male no. (%)	Female no. (%)	Male no. (%)	Female no. (%)
STI (HIV/AIDS)	23 (47.9)	25 (52.1)	28 (36.8)	48 (63.2)	99 (44)	126 (56)
Premarital sex	11 (57.8)	8 (42.2)	22 (28.2)	56 (71.8)	76 (56.3)	59 (43.7)
Unwanted pregnancy	8 (57.1)	6 (42.9)	28 (33.3)	56 (66.7)	76 (50.6)	74 (49.4)
Condom	22 (55)	18 (45)	15 (44)	29 (56)	55 (51.9)	51 (48.1)
SRH rights	22 (52.4)	20 (47.6)	21 (30)	49 (70)	74 (54.8)	61 (45.2)
Puberty	17 (42.5)	23 (57.5)	20 (32.3)	42 (67.7)	69 (59)	48 (41)

**Table 3:** Bivariate and multivariable binary logistic regression analysis for factors associated with parent-adolescent communication among high school students in Dabat town, northwest Ethiopia, 2018.

Variables	Parent- adolescent communication		COR (95% CI)	AOR (95% CI)
	Yes	No		
Age				
15–17	106	116	1	1
18-19	161	167	1.06 (0.75–1.48)	0.98 (0.68–1.40)

Sex				
Female	137	183	1	1
Male	130	100	1.74 (1.23–2.45)	**1.58 (1.1–2.25)** ^*∗*^

Religious participation				
At least once a week	172	183	1.67 (0.96–2.85)	1.4 (0.92–2.14)
At least monthly	56	75	2.09 (1.14–3.85)	0.68 (0.39–1.2)
Never	39	25	1	1

Family income				
<500	69	64	1	1
500–1,000	126	111	0.95 (0.62–1.54)	0.96 (0.62–1.49)
>1,000	72	108	1.617 (1.03–2.54)	**1.6 (1.02–2.57)** ^*∗*^

Knowledge of SRH				
Good knowledge	144	121	1.567 (1.12–2.19)	**1.47 (1.03–2.09)** ^*∗*^
Poor knowledge	123	162	1	1

Attitude towards SRH				
Favorable	177	138	2.06 (1.46–2.92)	**1.86 (1.29–2.67)** ^*∗*^
Unfavorable	90	145	1	1

^*∗*^Significant at 95% confidence interval; COR: crude odds ratio; AOR: adjusted odds ratio.

## Data Availability

All data generated or analyzed during this study are included in this article. The data that support the findings are also available from the corresponding and primary authors upon reasonable request.

## Abbreviations

AIDS: Acquired immune deficiency syndrome
SRH: Sexual reproductive health
STIs: Sexually transmitted infections
STDs: Sexually transmitted diseases
HIV: Human immune virus.
